# Innate local response and tissue recovery following application of high density microarray patches to human skin

**DOI:** 10.1038/s41598-020-75169-4

**Published:** 2020-10-28

**Authors:** David A. Muller, Joakim Henricson, S. Ben Baker, Totte Togö, Cesar M. Jayashi Flores, Pierre A. Lemaire, Angus Forster, Chris D. Anderson

**Affiliations:** 1 School of Chemistry and Molecular Biosciences, The University of Queensland, Building 76 Cooper road, St. Lucia, QLD 4072 Australia; 2 Department of Biomedical and Clinical Sciences, Linköping University, Linköping, Sweden; 3 Department of Emergency Medicine, Local Health Care Services in Central Östergötland, Linköping, Sweden; 4 Vaxxas Pty Ltd, Translational Research Institute, 37 Kent Street, Woolloongabba, QLD 4102 Australia; 5 Allergy Center Linköping, Region Östergötland, Sweden; 6 Division of Cell Biology, Faculty of Health Sciences, Linköping University, Linköping, Sweden

**Keywords:** Innate immunity, Vaccines

## Abstract

The development of microarray patches for vaccine application has the potential to revolutionise vaccine delivery. Microarray patches (MAP) reduce risks of needle stick injury, do not require reconstitution and have the potential to enhance immune responses using a fractional vaccine dose. To date, the majority of research has focused on vaccine delivery with little characterisation of local skin response and recovery. Here we study in detail the immediate local skin response and recovery of the skin post high density MAP application in 12 individuals receiving 3 MAPs randomly assigned to the forearm and upper arm. Responses were characterised by clinical scoring, dermatoscopy, evaporimetry and tissue viability imaging (TiVi). MAP application resulted in punctures in the epidermis, a significant transepidermal water loss (TEWL), the peak TEWL being concomitant with peak erythema responses visualised by TiVi. TEWL and TiVi responses reduced over time, with TEWL returning to baseline by 48 h and erythema fading over the course of a 7 day period. As MAPs for vaccination move into larger clinical studies more variation of individual subject phenotypic or disease propensity will be encountered which will require consideration both in regard to reliability of dose delivery and degree of inherent skin response.

## Introduction

**Figure 1:**
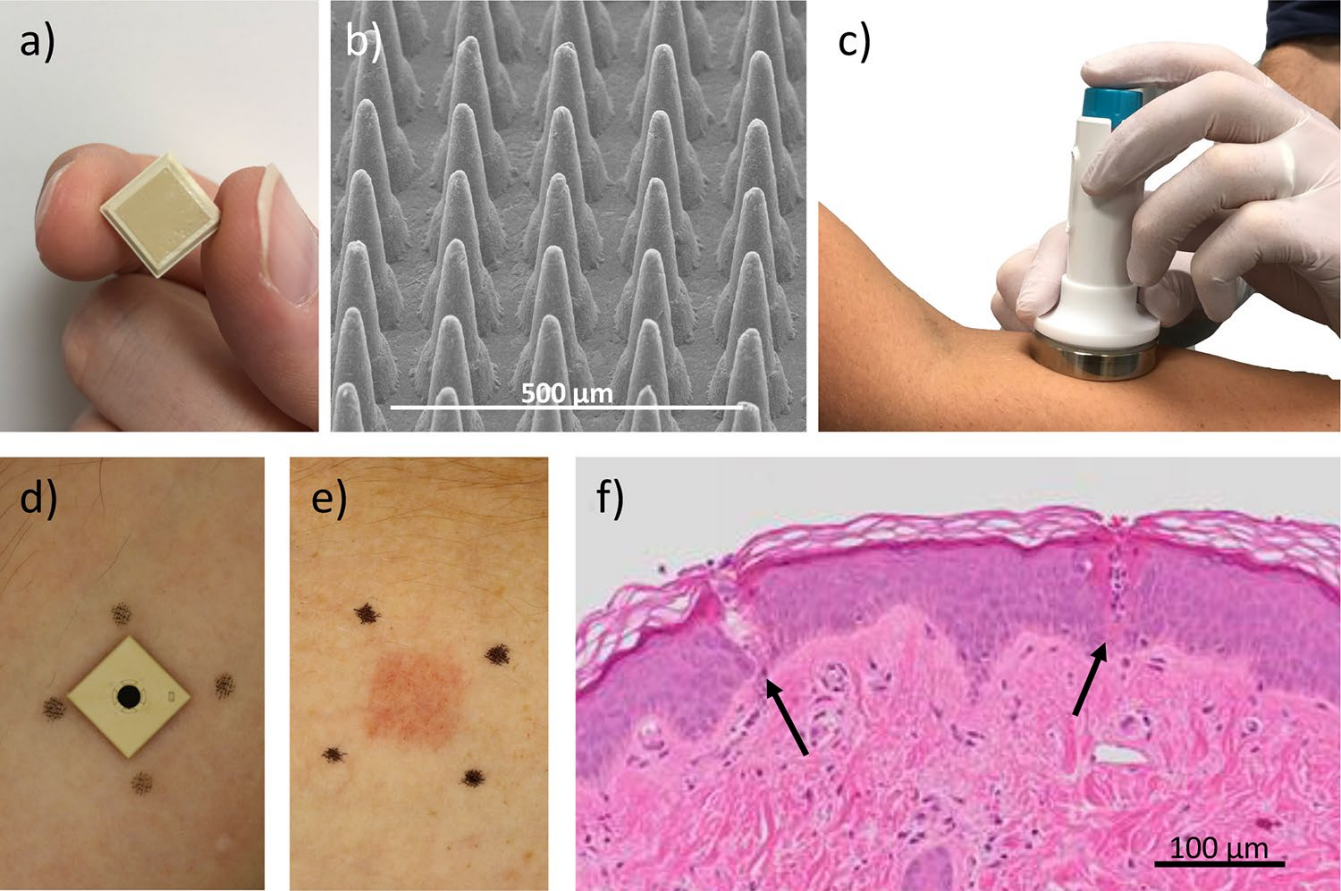
The HD-MAP and clinical application system (**a**) 1 × 1 cm polymer HD-MAP, (**b**) SEM image of uncoated HD-MAP. HD-MAP application to the (**c**) forearm by a spring-loaded applicator with skin conditioning ring (SCR) to improve HD-MAP engagement. (**d**) HD-MAP applied to the skin, (**e**) representive image of the erythema reponse following removal of the HD-MAP applied to forearm skin (**f**) representative histology slide showing the puncture tracks following HD-MAP removal.

The skin is a protective barrier to the outside world with innate reactive capability. This capability is the result of an evolutionary development of complex protective immune and inflammatory responses to an assortment of common, benign or pronounced, provocations including ultraviolet radiation, toxins, viral or bacterial pathogens and minimal mechanical wounding^1–8^. This protective, inflammatory and immunological capability makes the skin an attractive target for efficient vaccine delivery. It has been well documented there are many advantages to intradermal (ID) vaccine delivery compared to intramuscular and subcutaneous injections, such as, enhanced immune responses and fractional dosing (ie dose sparing of vaccine)^9–12^. However, use of a traditional needle and syringe ID injection can often depend on a cold chain, requires a skilled operator and is reasonably invasive. As an alternative approach, microarray patches (MAPs) delivered by a less medically skilled operator can reproducibly deliver vaccine into the dermal and epidermal layers of the skin that contain high densities of immune cells^13^. Currently, MAPs for vaccine delivery are being developed in a range of configurations: from low density dissolving arrays to high density solid array patches. The MAP used in the current investigation, is a solid, injection-moulded polymer, 1 cm × 1 cm square, with a high density microprojection array (HD-MAP) (Fig. 1a) containing 3,136 projections 250 µm in length (Fig. 1b) onto which vaccine formulation can be coated.

Microneedle arrays have a wide range of uses from cosmetic^14,15^ (e.g. Dermaroller™), diagnostic^16,17^, drug delivery^18–20^ through to the most promising and most studied area, vaccine delivery^21^. In small animal models comparative dose matched studies with standard injection methods, HD-MAPs have routinely been shown to produce enhanced immune responses with enhanced kinetics of IgG induction with a fractional dose compared to needle and syringe for a wide range of vaccines from inactivated virus^22,23^, split virus^24^, conjugated^25^, DNA^26^ and virus like particle vaccines^27^. This enhanced response has largely been attributed to the induced cell death around each projection causing a mixture of damage and pathogen (vaccine) danger signals ^28^. It has been shown that these enhanced immune responses can be further boosted by the addition of adjuvants which have a synergistic effect when combined with HD-MAP vaccine delivery^29^. While these immunogenicity phenomena have been extensively studied in preclinical models the immunogenicity benefits are yet to be demonstrated in humans. In addition to the potential immunological benefit of HD-MAPs, there is the possibility to remove or reduce the dependency on the cold chain since some dried vaccines have been shown to have excellent stability profiles at higher temperatures^30^.

Although there is a large body of research in small animals, the use of MAPs for human vaccination is currently the subject of only a handful of clinical trials^13,31–34^. Work previously reported by our co-authors using a solid silicon microarray patch, called the Nanopatch, applied to the skin with a similar applicator system found MAP application resulted in an immediate mild erythema and oedema response which lasted for a period of days. The focus of these papers was on safety and immunogenicity (in phase 1 influenza trials) and the clinical features associated with use of the MAP^13^. Our paper seeks to elucidate in detail the various aspects of skin reactivity.

Each HD-MAP application results in a regular array of up to 3,136 micropunctures in the skin (Fig. 1f). While several studies have been performed on low density MAP arrays^35–37^, no detailed studies have been published investigating the skin response and the healing kinetics of the micropunctures and return of the skin’s barrier function following high-density MAP application for vaccination. Such information would inform on reliability and variability of both penetration of the skin (implying successful vaccine delivery), induction of response (implying damage response effect) and recovery (relevant for subject acceptability) as well as being per se an illustration of an individual’s capability for innate skin reactivity.

In this study, we applied sterile, excipient-coated MAPs to the forearm and upper arm of volunteers using a spring-loaded applicator (Fig. 1c). After 2 min wear time (Fig. 1d) the MAP was removed (Fig. 1e), this wear time having been used in previous clinical studies with high density arrays, resulting in required vaccine delivery^13^. The erythema reaction in the skin (Fig. 1e) was quantified by tissue viability imaging (TiVi) (Fig. 1f). The loss of water barrier function following HD-MAP application was quantified by transepidermal water loss (TEWL). In addition to TEWL and TiVi, the HD-MAP application site was visually documented by dermatoscopy. The course of the skin reaction recovery was followed over a 7-day period.

## Results

### Clinical assessment of HD-MAP application

**Figure 2:**
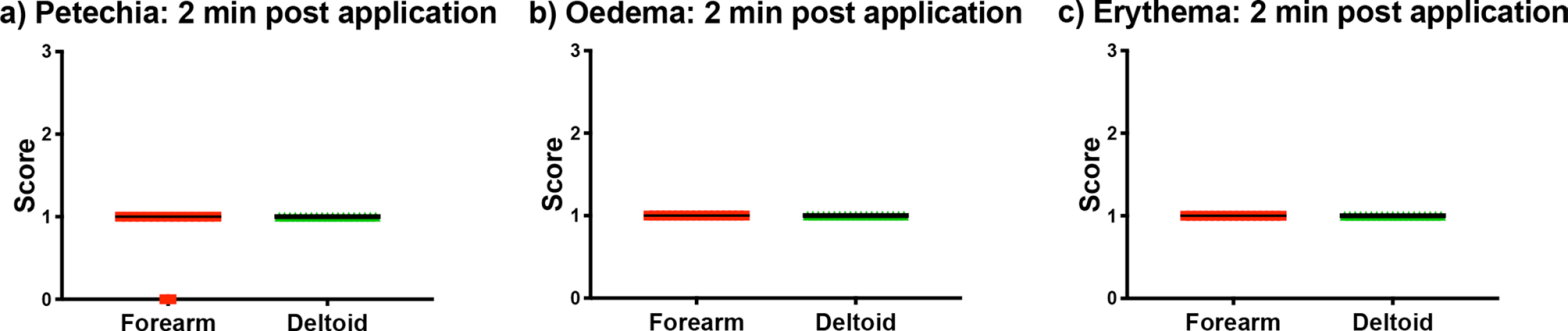
Clinical assessment of (**a**) petechial, (**b**) oedema and (**c**) erythema. Line at medium.

As in previous publications^13,32^, classical clinical scoring by eye was performed by a trained clinical staff. Erythema, oedema and the presence of petechia were scored on a scale of 0–3. Minor reactions were recorded with all subjects being scored 1 for erythema, oedema and petechia (Fig. 2a–c) at both upper arm and forearm sites. In addition to the clinical scoring, patch sites were assessed for minimal break through or “wet” bleeding, as evidenced by the presence of multiple pin point blood spotting on an applied tissue paper. This was seen in 10 of 36 HD-MAP applications (28%) immediately upon removal, but at no subsequent time point. These clinical observations are similar to those recorded in previous studies from our group^7, 26^.

### Photography of HD-MAP application sites

**Figure 3:**
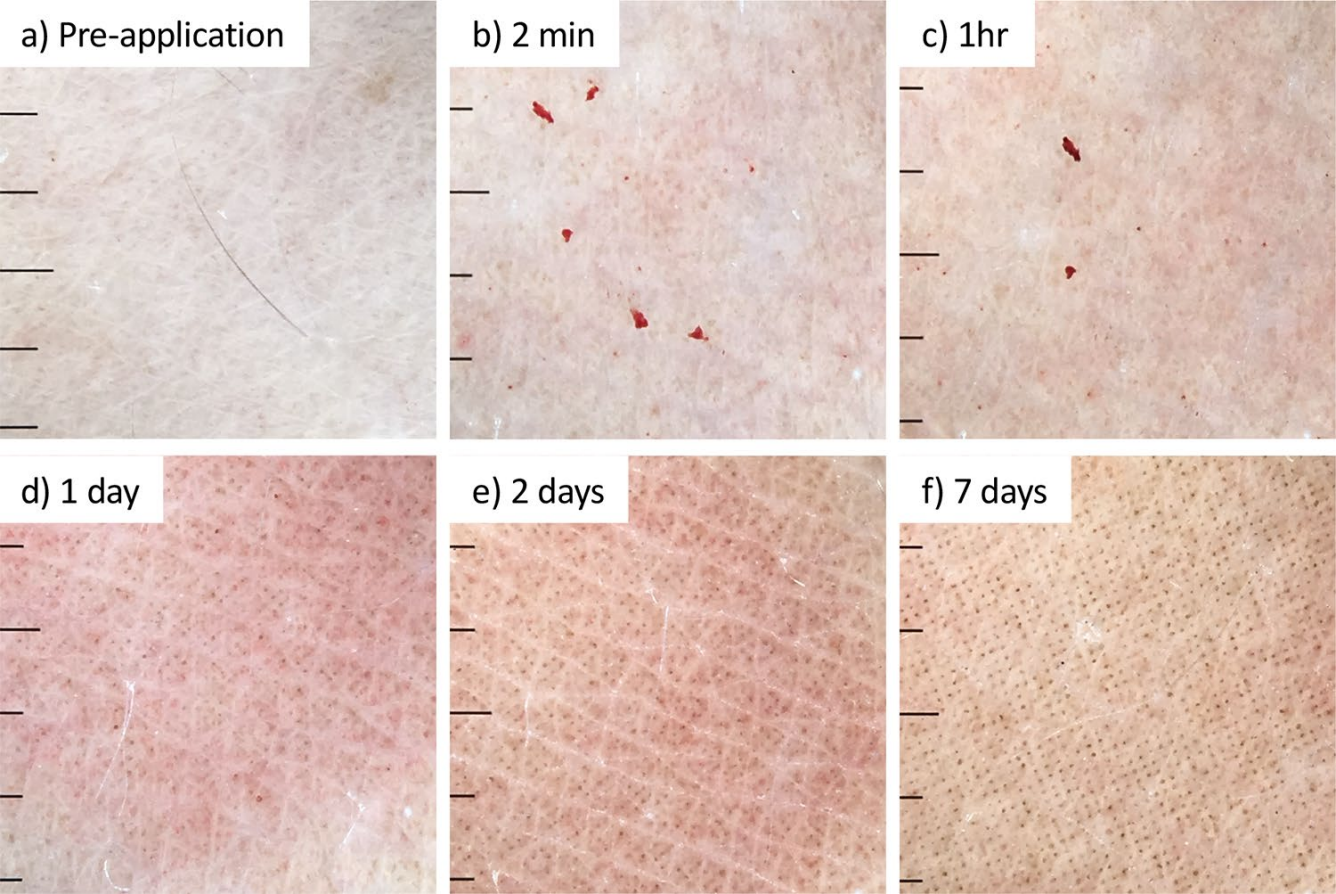
Representative dermatoscopy imaging of HD-MAP applications sites demonstrating the skins reaction, recovery and appearance of points of discolouration corresponding to engaged HD-MAP projections at (**a**) pre-application, (**b**) 2 min, (**c**) 1 h, (**d**) 1 day, (**e**) 2 days and (**f**) 7 days. Each scale bar interval = 1 mm.

Dermatoscopic imaging provided a convenient data set with which to document skin reactivity. Figure 3, is a representative image set. Prior to HD-MAP application the skin was normal, with damaged skin, moles, tattoos or abrasions having been avoided (Fig. 3a). Immediately after HD-MAP removal (Fig. 3b) the erythema response had begun with the skin becoming red directly under the site of HD-MAP application. Initially, after HD-MAP removal petechiae was observed as a common feature (91% of applications). The petechiae were still visible at 1 h (Fig. 3c) but were not seen at 24 h (Fig. 3d). At 24 h post MAP application a regular array of points of dark discolouration (unrelated to erythema) began to appear at varying degrees of intensity. The points corresponded to the periodicity of the microprojections of the HD-MAP (Fig. 3d). These spots increased in intensity (Table 1) over the course of the study (Fig. 3e,f). While not investigated in this manuscript it is tempting to speculate that the number of spots could be used as a surrogate for HD-MAP engagement and potentially vaccine delivery. Table 1 compares the degree and chronology of the observed petechiae and points of discoloration analysed post hoc.

**Table 1:** The number of HD-MAP application sites with petechia and/or points of discolouration (corresponding to engaged HD-MAP projections) scored based on semi-quantitative post hoc analysis of dermatoscope images.

Time	Petechia				Points of dark discolouration corresponding to engaged HD-MAP projections			
	Mild	Moderate	Severe	Not observed	Mild	Moderate	Well defined	Not observed
Pre	0	0	–	36	–	–	–	36
2 min	30	3	–	3	–	–	–	36
10 min	33	3	–	0	1	–	––	35
60 min	32	1	–	3	1	–	–	35
120 min	26	3	–	7	2	–	–	34
1 day	0	0	–	36	23	6	1	6
2 days	0	0	–	36	23	10	1	2
7 days	0	0	–	36	11	16	6	3

### Quantitative assessment of skin reactivity by TiVi and TEWL

**Figure 4:**
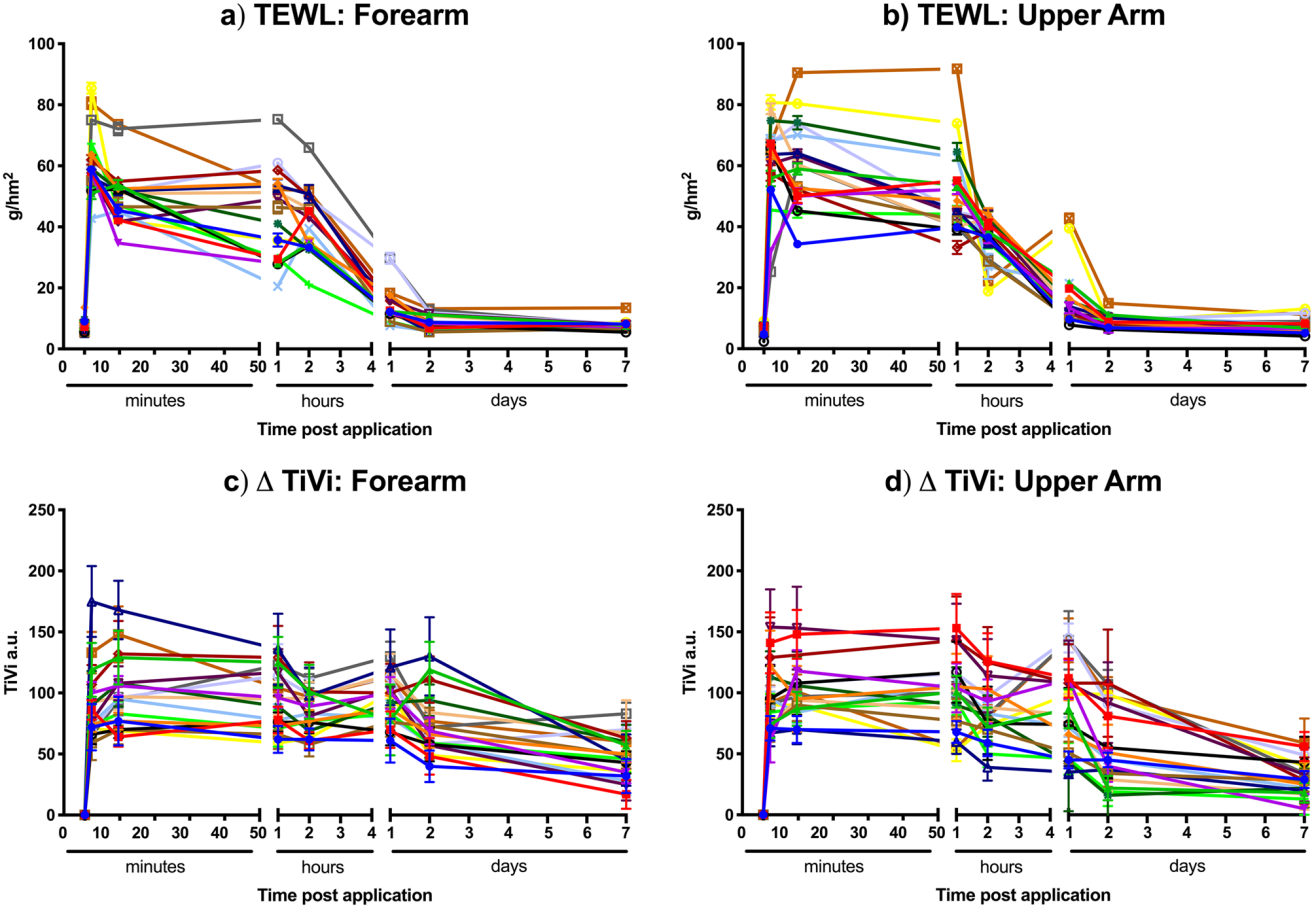
Skin reactivity and recovery post HD-MAP application. Measurement of transepidermal water loss from (**a**) the forearm or (**b**) upper arm for each HD-MAP application. Erythema was quantitated by TiVi imaging of HD-MAP application sites to the (**c**) forearm or (**d**) upper arm for each HD-MAP application.

In this study we observed baseline TEWL measurements ranging from 5 to 14 g/hm^2^ (subject dependent). As seen in Fig. 4a,b, all 12 subjects at all application sites experienced a significant increase in transepidermal water loss peaking within the first 10 min when the barrier function of the skin had been perturbed by the HD-MAP application with water loss increasing to 50–100 g/hm^2^. Again, the actual level of flux and overall increase was subject dependent on both forearm and upper arm sites. From the initial peak, the TEWL signal decreased over the next 48 h when the TEWL response returned to baseline. All subjects showed similar responses in terms of kinetics/return to baseline.

Erythema responses were calculated for all HD-MAP application sites with the TiVi imaging system (Fig. 4c,d). Here a rapid erythema response was observed which was visible from HD-MAP removal, this response peaked between 2 and 10 min. The induced erythema response slowly dissipated over the course of the 7-day observation period. In some subjects, erythema was still observed at 7 days while others had returned to baseline values. The subject erythema response was largely subject dependant with several subjects having a second peak in redness 48 h post application.

### HD-MAP engagement

**Figure 5:**
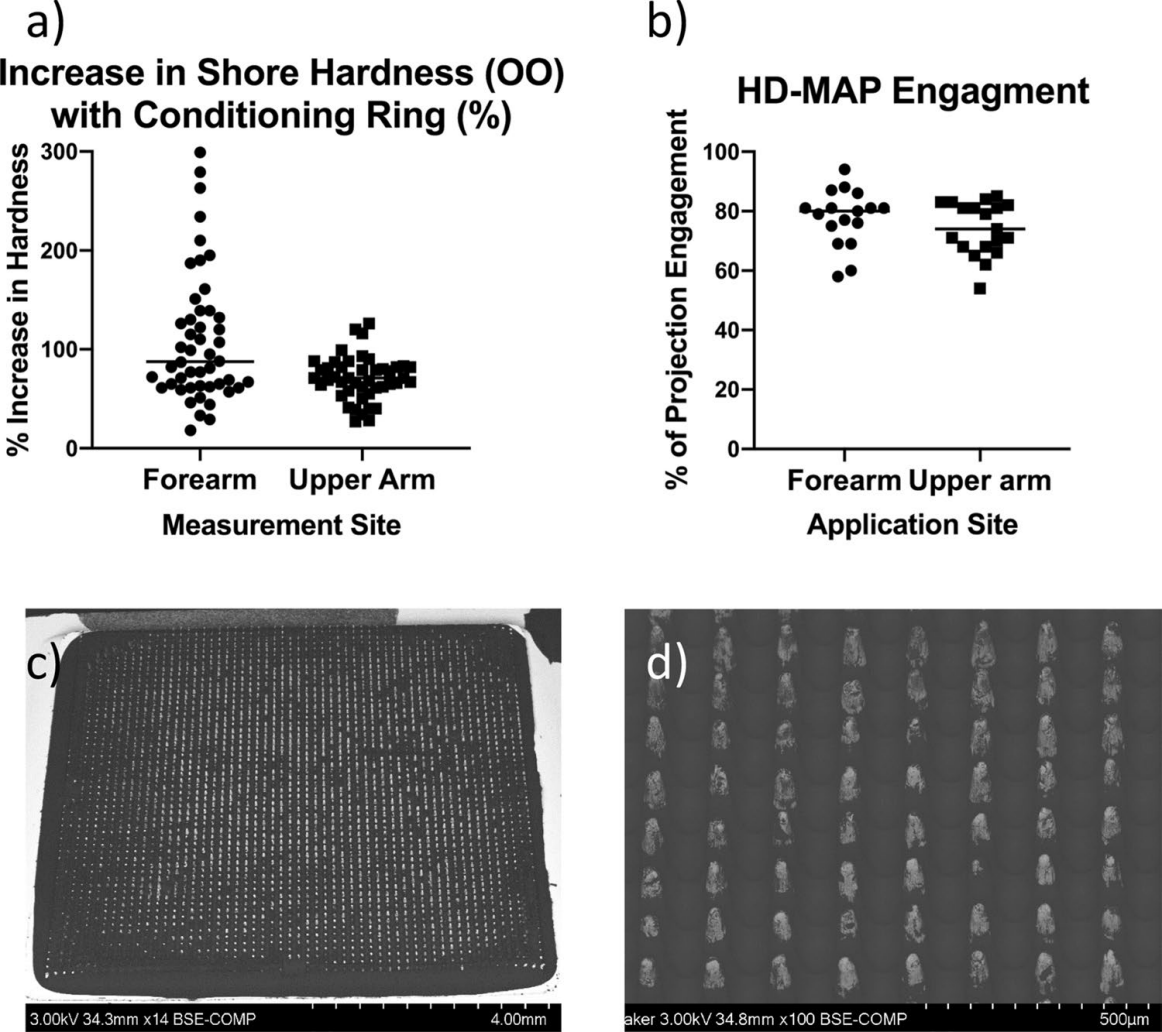
(**a**) Percentage increase of skin hardness at HD-MAP application sites when conditioned by the SCR. (**b**) Percentage engagement of each HD-MAP application on the upper arm and forearm. Following application HD-MAPs were analysied for removal of the coating (white areas) by SEM, a representive HD-MAP (**c**) overview is shown and (**d**) close up image. Error bars represent ±  standard deviation of the mean.

Data gathered in previous studies have shown HD-MAP application to uncompressed skin results in relatively lower immunogenicity titres for upper arm application compared to the volar forearm^13^. This may have been linked to decreased engagement of the HD-MAP with the skin and the more variable upper arm site. In our study therefore, HD-MAP application was performed in conjunction with a skin conditioning ring (Supplementary Videos 1 and 2) to apply a compression force which tensions and slightly raises the skin for HD-MAP delivery. Using skin hardness assessment, we could show that this manouver effectively increased skin hardness (Fig. 5a). Following the application, the HD-MAP was removed from the skin and stored for subsequent SEM analysis (Fig. 5c,d). This analysis allowed for the visualisation of the removal of the HD-MAP coating and thus the determination of the percentage of projections that entered the skin. Using this application condition with a 2-min wear time, resulted in consistent engagement across both applications sites with an average of 78% ± 9% and 74% ± 9 HD-MAP engagement on the forearm and upper arm respectively (Fig. 5b).

## Discussion

The World Health Organisation has identified MAPs as a potential game-changer for vaccine distribution and coverage in low-to-middle income countries^38^. Given the potential for MAPs to become the vaccine delivery method of choice, understanding how the MAP itself interacts with the skin is important knowledge. Here we present a study evaluating in detail the reactivity and recovery of skin following high density polymer MAP applications to the forearm and upper arm. We documented and quantified the recovery process by adding dermatoscopy, TEWL, and TiVi to traditional clinical scoring methods. To execute the study, 12 subjects were recruited, each receiving a total of 3 HD-MAPs across both the volar forearm and the upper arm. Reactivity and recovery from these HD-MAP applications were investigated in detail.

An application sensation, erythema, formation of petechia and oedema after the HD-MAP application was ubiquitous^32^, but of low degree and individually variable in the healthy volunteers studied. We interpret the reactivity seen as evidence of an innate reactivity to minimal trauma illustrating both the capability of the HD-MAP to deliver a vaccine to the skin and incite an inflammatory reaction. This inflammatory reactivity is likely essential for the enhanced immunogenicity which has been demonstrated in vaccination studies^28^. Additionally, the HD-MAP provocation is per se a convenient method for demonstration of the skin’s innate reactivity in general terms. In a broader vaccination population, an individual variability may be more pronounced in subgroups with skin sensitivity.

Traditionally, MAPs have been advanced as a vehicle for “pain free” vaccination. Our observations, and those of Fernando et al.^13^ and others^33,34^ show that tissue reaction is an inevitable (innate) result of even uncoated microneedle application and can be expected to be more pronounced with the delivery of vaccines, excipients or other materials into the skin. We view the inflammatory response observed in subjects in this study as a central mechanism for the demonstrated ability of HD-MAP delivered vaccines to enhance immunological efficacy. To maximise this effect and to better understand possible variability in response in a broader vaccination population further study is warranted, including use of methods characterising the oedema component of reactivity. In this regard, the interaction between sex, age, application site, ethnic backgrounds, and reactive variability are of interest. Quantitative assessment methods will allow demonstration of individual variability.

A surprising finding was the dermatoscopic demonstration of points of discolouration on the skin corresponding spatially to the microprojection pattern on the HD-MAP (Fig. 3e). While this appeared to coincide with the return to baseline of the TEWL measurements no mechanistic connection can at the moment be assigned. Currently, the nature of these points of discolouration remains undetermined, however we hypothesise that they might be dried or oxidised tissue exudates or other discolouration. The cause and composition of these spots are currently under investigation.

Polarised reflectance spectroscopy, TiVi, was used to assess and quantify the resulting erythema following multiple HD-MAP applications to subjects. In all subjects examined the TiVi index rapidly increased from baseline following HD-MAP application. This increase in TiVi index is directly proportional to the concentration of red blood cells (RBC) at the application site. In parallel to the increased RBC concentration it is thought the HD-MAP application produces an inflammatory state within the skin. The dynamic application of the HD-MAP to the skin at 20 m/s causes a rapid release of damage-associated molecular patterns (DAMPs) along with a rapid increase in cellular infiltrate. Although, outside the scope of the current study, previous studies in mice have suggested that the colocalization of DAMP signals produced during cell death with vaccine formulations are partly responsible for the increased immune responses observed in small animal models^28^. Following the initial increase in erythema immediately after HD-MAP application, the erythema signal slowly degraded over the course of 7 days. Each HD-MAP reacted independently of other applications with erythema contained within the boundaries of the HD-MAP with minimal spreading observed. Although our findings have clear relevance to HD-MAP application it is limited to placebo coated HD-MAPs. Additionally the study is limited to Fitzpatrick skin types 1–3, however further studies are currently under investigation for the Fitzpatck skin types 4–6 and potential vaccination subgroups in the populations such as the elderly.

TEWL is a classic method for assessing the barrier function in skin. Water loss from the skin was a ubiquitous sign of a loss of barrier function, in this case produced from successful HD-MAP engagement. While there is always a background level of water vapour escaping the skin, once the skin is punctured by the HD-MAP the integrity of the stratum corneum is lost and rate of water loss from the viable epidermis increases markedly. However, regardless of the magnitude of the response, all HD-MAP application sites saw a return to baseline by 48 h after HD-MAP application. This suggests a return of the skin’s barrier function, as a part of normal wound healing kinetics after the minor trauma caused by the HD-MAP application. Previous work by Gupta et al. using a low-density stainless steel MAP found water loss returned to baseline within 2 h of HD-MAP application^39^. The difference between the results by Gupta et al. and those presented in this study is likely due to the geometry and projection density of the MAPs used or sensitivity of the use evaporimetor. Furthermore, Gupta et al. used a 50 projection microneedle array (500–1500 µm × 75 µm thick × 200 µm wide) which would likely slice into the skin versus the MAP used in the current study which causes 1000 s of micro-punctures through the stratum corneum into the viable epidermis and papillary dermal layers of the skin.

In the context of vaccine delivery, 100% MAP engagement is desirable though not essential for the required vaccine dose to be delivered. Consistent and reliable MAP engagement is, however, essential to ensure that reliable and effective vaccine doses are administered by the HD-MAP. To this end, a dynamic application of HD-MAPs at 20 m/s is required to penetrate the stratum corneum and enter the underlying dermal and epidermal layers of the skin. Using this dynamic application system coupled with the skin conditioning ring gave, in the present study, consistent HD-MAP engagement at both the forearm (78% ± 9) and upper arm (74% ±  9) application sites to a depth of approximately 90 µm. These engagement levels are known to be consistent with adequate dose delivery. As stated above, this application is responsible for the marked increase in erythema and TEWL. Additionally, the patch application can rupture capillaries at the site of application resulting in transient petechia at both forearm and upper arm sites. Although these finely distributed petechia are observed via dermatoscopy for each application, the number of petechia does not correspond directly to the number of projections, which we interpret as evidence that details of the microcapillary bed anatomy influence the occurrence of petechiae.

In conclusion, further detailed studies characterising HD-MAP interaction with the skin need to be performed to determine suitability for use and risk of morbidity over a broader range of recipient populations. We have demonstrated the application of HD-MAPs results in skin injury with different indicators of injury resolving at different time scales. Reactivity to the minimal trauma can be expected to show individual variability, as has been shown for other types of innate reactivity such as inflammation caused by UVB and irritation. Since the evidence points to the fact that a prerequisite of the physical adjuvant effect is an adequate degree of inflammatory response, quantification of individual variability in health and disease can be expected to be important.

## Materials and methods

### HD-MAP fabrication

HD-MAPs were produced by injection moulding medical-grade synthetic polymer to produce microprojection arrays 250 µm in length and 5,000 projections/cm^2^. To facilitate improved imaging, in this case, HD-MAPs were sputter-coated with gold and before being coated with 1% w/v Hypromellose (Shin-Etsu Chemical Company Ltd, Japan) 0.72% w/v trehalose (Pfanstiehl, Germany) and dPBS (Sigma Aldrich, USA) using methods previously described by Griffin et al.^32^. Coated MAPs were sealed in aluminium medicans (Amcor, UK) and gamma sterilised (≥ 25 kGy, Steritech, Australia). The gold and placebo excipient coatings allowed for post-application inspection of projection penetration (loss of placebo coating) using backscatter scanning electron microscopy (SEM) (Centre for Microscopy and Microanalysis, University of Queensland).

### HD-MAP application

All HD-MAP applications were performed by experienced personnel. Prior to HD-MAP application, sites on the upper arm (Supplementary Video 1) and forearms (Supplementary Video 2) were selected and marked using a template and cleaned with an alcohol swab. A skin conditioning ring was placed over the application site and an applicator, preloaded with the HD-MAP was docked within the skin conditioning ring. The skin ring, pre-calibrated to apply a force of approximately 30 N, was pressed down to pretension the skin, to approximately 30 N then the applicator was triggered to apply the HD-MAP at a speed of 25 ms^−1^. After application the applicator and skin conditioning ring were removed and the HD-MAP was left in place, with projections embedded in the skin, for 2 min. Following the 2-min wear time the HD-MAP was removed from the upper arm or forearm by directly lifting the HD-MAP up while manually tensioning the skin (Supplementary Videos 3 and 4 respectively). The removed HD-MAP was then stored for subsequent analysis of projection engagement and penetration into the skin.

### Skin hardness measurement

To quantify the effect the skin conditioning ring imparted on the skin, hardness measurements were collected pre-application. A durometer was used to take shore hardness measurements (OO scale) at each HD-MAP application site. A measurement was first done with the SCR in place, but with no downward pressure applied, and then again with the SCR exerting a 30 N downward force on the skin.

### Histological analysis

Punch biopsies (4 mm, ProSciTech, Australia) collected by a Dermatologist, were taken from the volar forearm following HD-MAP application for histological analysis. The biopsy sites were stitched with dissolving suture material following removal of the biopsies. The skin biopsies for histology were fixed in a 10% neutral buffered formalin solution. Sections were stain with Haematoxylin and Eosin (H&E, Haematoxylin, Signa-Aldrich, USA; Eosin Y, ProSciTech, Australia. Paraffin-embedded H&E stained tissue samples were scanned on the Olympus VS120 slide scanner using Olympus VS-ASW software (version 2.9), with a 20 × air objective (Olympus).

### Clinical scoring

Erythema, oedema, and petechiae were scored on a 0–3 scale representative of none, mild, moderate, or severe, relative to untreated skin, as previously described by Fernando et al.^13^ and Griffin et al.^32^.

### Transepidermal water loss measurement from HD-MAP application sites

Transepidermal water loss (TEWL) measurements were taken using a Tewameter-300 probe attached to a MDD4 driver unit (Courage-Khazaka)^40^. Measurements were taken at intervals described in the study design on subjects who had equilibrated to the testing room environment for at least 30 min. The Tewameter probe was placed on the test site using a protective sticker on the rim of the chamber to avoid direct skin contact with the probe. Measurements were collected every second for 30 s and stabilised values selected for analysis^32^.

### Measurement of erythema responses at HD-MAP application sites

The red blood cell (RBC) concentration (erythemal response) at the HD-MAP application site was quantified using reflectance polarisation spectroscopy, Tissue Viability Imaging (TiVi, Wheels Bridge AB), described in detail by O’Doherty et al.^41^. Images were captured in duplicate before MAP application in the absence of ambient light and at various intervals post-application as outlined in the study design. Images were analysed in the associated TiVi software (wheelsbridge.se), with erythema determined by selecting the HD-MAP application site (excluding surrounding skin) and the intensity of the erythema response quantified.

### Polarised light high-resolution photography

Examination of the HD-MAP application site was documented by polarised light high-resolution photography at time intervals described in the study design. Using a iC1 Dermatoscope (Heine) attached to an iPhone 6 (Apple), images of the MAP application site were captured at maximum optical zoom as another method by which to assess MAP engagement, skin response, and recovery of the skin.

### SEM analysis and MAP engagement calculation

After application, MAPs were stored for imaging with a scanning electron microscope (Hitachi SU3500) in backscatter mode. This allowed the determination of the proportion of projections from which the placebo coating had been removed, allowing the gold coating to be visualised. This was interpreted as evidence of projection penetration into the skin which reflects delivery of the coating, ImageJ software (National Institutes of Health) was used for image analysis.

### Ethics

The study “Minimally invasive studies of the skin’s innate reactivity—microneedle provocation” was approved by the Linköping Ethical Board (MB 2017-409-31). The study described has been carried out in accordance with the code of ethics of the world medical association (declaration of Helsinki) for experiments involving humans. Written informed consent was obtained from each subject participating in the study.

### Study design

Twelve participants (6 male and 6 female, aged 18–65 years old; Table S1) were recruited according to our inclusion/exclusion criteria (see Suspplementary information, S2). Subjects visited the Skin Laboratory a total of 5 times. Initially, baseline measurements were recorded 2 days prior to HD-MAP application with TiVi, TEWL, and photography at application sites. Preliminary studies determined the optimal time points for assessment of the skin response to HD-MAP application (data not shown). On the HD-MAP application day subjects received 3 HD-MAPs in total, randomised between dominant and non-dominant arms with, at a study level, equal numbers of HD-MAPs applied to either the forearm or upper arm. HD-MAPs were all left in place on the skin for 2 min, after which TEWL, TiVi and application site photographs were recorded at 2, 10, 60 and 120 min, then follow-up at 24 h, 48 h and 7 days. HD-MAPs were applied to normal, healthy skin, avoiding areas with scars, excessive hair, tattoos, and damaged/diseased skin. A demonstration of the mechanics of MAP application was given to the subject. All HD-MAPs were stored for subsequent SEM analysis of degree of engagement.

### Statistical analysis

All statistical analysis was performed in GraphPad Prism for Mac OS X version 7.0c (https://www.graphpad.com).

## Supplementary information

Supplementary Information 1.

Supplementary Video 1.

Supplementary Video 2.

Supplementary Video 3.

Supplementary Video 4.
