# Supplementary material for: Innate local response and tissue recovery following application of high density microarray patches to human skin
Source: Sci Rep. 2020 Oct 28;10:18468. doi: 10.1038/s41598-020-75169-4 (PMC7595201; doi:10.1038/s41598-020-75169-4)
Supplement: Supplementary file 1 — Supplementary Information 1. [file 41598_2020_75169_MOESM1_ESM.pdf]

Innate local response and tissue recovery following application of high-density microarray patches to human skin.

Authors
David A. Muller[1*], Joakim Henricson[2,3], S. Ben Baker[4], Totte Togö[5] Cesar M. Jayashi[4], Pierre A. Lemaire[4], Angus Forster[4], Chris D. Anderson[2,6*]

Affiliations
[1] School of Chemistry and Molecular Biosciences, The University of Queensland, Building 76 Cooper road, Queensland 4072, Australia.
[2] Department of Biomedical and Clinical Sciences , Linköping University, Linköping, Sweden.
[3] Department of Emergency Medicine, Local Health Care Services in Central Östergötland, Sweden.
[4] Vaxxas Pty Ltd, Translational Research Institute, 37 Kent Street, Woolloongabba, Queensland 4102, Australia.
[5]Allergy Center Linköping, Region Östergötland, Sweden
[6] Division of Cell Biology, Faculty of Health Sciences, Linköping University, Sweden.

*To whom Correspondence may be addressed
Dr David Muller
Postal Address: School of Chemistry and Molecular Biosciences, Cooper Road
The University of Queensland,
St Lucia, 4072, Queensland, Australia

Email: d.muller4@uq.edu.au

Prof Christopher Anderson
Email: chris.anderson@regionostergotland.se

Supplementary Information

Table s1: Patient information.

**Patient ID**	**Gender**	**Age**
101	Male	41
102	Male	28
103	Male	52
104	Female	20
105	Male	20
106	Female	46
107	Female	48
108	Male	23
109	Female	36
110	Male	35
111	Female	20
112	Female	20

Supplementary information S2: Inclusion and Exclusion Criteria

Inclusion criteria:
Voluntary participation
Between 18 and 55 in age
Expected to have normal skin function and reactivity
Generally healthy

Exclusion critera:
Do not understand the written and oral information
Do not sign the written consent form
Previous or ongoing skin disease(s) or skin problems
Previous experience of or ongoing altered function and healing of the skin

Supplementary video s1: HD-MAP application to the upper arm

Supplementary video s2: HD-MAP application to the forearm

Supplementary video s3: HD-MAP removal from the upper arm

Supplementary video s4: HD-MAP removal from the forearm
